# Mitochondrial Respiration - An Important Therapeutic Target in Melanoma

**DOI:** 10.1371/journal.pone.0040690

**Published:** 2012-08-17

**Authors:** Michelle Barbi de Moura, Garret Vincent, Shelley L. Fayewicz, Nicholas W. Bateman, Brian L. Hood, Mai Sun, Joseph Suhan, Stefan Duensing, Yan Yin, Cindy Sander, John M. Kirkwood, Dorothea Becker, Thomas P. Conrads, Bennett Van Houten, Stergios J. Moschos

**Affiliations:** 1 Department of Pharmacology and Chemical Biology, University of Pittsburgh, Pittsburgh, Pennsylvania, United States of America; 2 Department of Medicine, University of Pittsburgh, Pittsburgh, Pennsylvania, United States of America; 3 Gynecologic Cancer Center of Excellence, Women's Health Integrated Research Center at Inova Health System, Annandale, Virginia, United States of America; 4 Department of Biostatistics, University of Pittsburgh, Pittsburgh, Pennsylvania, United States of America; 5 Department of Biological Sciences, Carnegie Mellon University, Pittsburgh, Pennsylvania, United States of America; 6 Sektion Molekulare Uroonkologie, Urologische Universitätsklinik Heidelberg, Heidelberg, Germany; 7 Department of Pathology, University of Pittsburgh, Pittsburgh, Pennsylvania, United States of America; University of Medicine and Dentistry of New Jersey, United States of America

## Abstract

The importance of mitochondria as oxygen sensors as well as producers of ATP and reactive oxygen species (ROS) has recently become a focal point of cancer research. However, in the case of melanoma, little information is available to what extent cellular bioenergetics processes contribute to the progression of the disease and related to it, whether oxidative phosphorylation (OXPHOS) has a prominent role in advanced melanoma. In this study we demonstrate that compared to melanocytes, metastatic melanoma cells have elevated levels of OXPHOS. Furthermore, treating metastatic melanoma cells with the drug, Elesclomol, which induces cancer cell apoptosis through oxidative stress, we document by way of stable isotope labeling with amino acids in cell culture (SILAC) that proteins participating in OXPHOS are downregulated. We also provide evidence that melanoma cells with high levels of glycolysis are more resistant to Elesclomol. We further show that Elesclomol upregulates hypoxia inducible factor 1-α (HIF-1α), and that prolonged exposure of melanoma cells to this drug leads to selection of melanoma cells with high levels of glycolysis. Taken together, our findings suggest that molecular targeting of OXPHOS may have efficacy for advanced melanoma.

## Introduction

Despite the recent US Food and Drug Administration (FDA) approval of novel therapies for advanced melanoma, the prognosis for locally advanced and stage IV melanoma remains poor because of emerging resistance to molecular therapies, and the relatively low number of patients with metastatic melanoma who benefit from immunotherapies [1], [2]. Thus, it is essential to further identify signaling pathways and cellular processes that are pertinent regulators of melanoma progression and advanced melanoma. We herein present novel and important data, which show that cellular bioenergetics and, in particular, mitochondrial functions play an important role in this disease.

Involvement of pro- and anti-apoptotic mitochondria-associated proteins in melanoma cell survival has previously been described [3]–[5]. However, to date, little is known regarding the role of mitochondrial functions, such as redox regulation and OXPHOS, in melanoma progression and survival. A previous study, which investigated redox regulation in melanoma progression focused on the physicochemical properties of melanin as an anti-oxidant or a pro-oxidant [6]. These mitochondrial functions are linked because oxygen levels affect the dependence of cells on OXPHOS for energy production and the production of reactive oxygen species (ROS). The other important question that has not yet been systematically addressed is whether melanoma cells rely more on OXPHOS or glycolysis [7], [8].

The drug Elesclomol has been shown to alter redox balance in cells, and to act as a strong inducer of oxidative stress [9]. In preclinical models it was found to enhance the cytotoxic effects of the chemotherapeutic agent, paclitaxel [10]. Furthermore, significant progression-free survival (PFS) benefit for metastatic melanoma was observed in a small randomized phase II trial of Elesclomol combined with paclitaxel versus paclitaxel alone [11]. A large randomized phase III study of Elesclomol plus paclitaxel versus paclitaxel alone was further conducted in patients with metastatic melanoma [12]. In this study, preplanned subgroup analysis of patients with normal lactate dehydrogenase (LDH) versus patients with high serum LDH levels, a known adverse prognostic factor for patients with metastatic melanoma [13], suggested that the combination of Elesclomol and paclitaxel compared with paclitaxel alone significantly prolonged median PFS only in patients with metastatic melanoma and normal serum LDH. These data may be an indication that oxidative stress-associated cellular processes are affected in patients with normal levels of serum LDH.

Using Elesclomol as a tool to study oxidative stress-associated cellular processes in cells representing advanced melanoma, we demonstrate that the drug alters the abundance of proteins involved in OXPHOS. Furthermore, our analyses focusing upon bioenergetics revealed that Elesclomol inhibits OXPHOS without a major effect on glycolysis, and that melanoma cells have higher OXPHOS activity compared with human epidermal melanocytes.

## Results

### Elesclomol treatment increases ROS in melanoma cells in non-melanosomal structures

**Figure 1:**
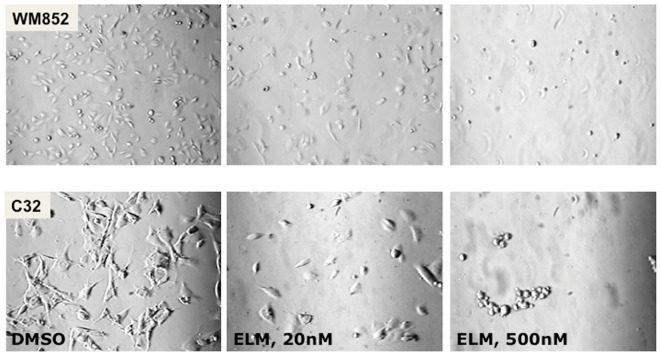
Phase-contrast analysis of Elesclomol-treated melanoma cells. Phase-contrast images of a pigmented (WM852) and an amelanotic (C32) melanoma cell line treated for 12 hr with drug vehicle (DMSO), or a low (20 nM), or high dose (500 nM) of Elesclomol (ELM). (Images were captured at 20× magnification).

It has been previously reported that Elesclomol treatment increases ROS in melanoma and other cancer cells, an observation we confirmed in WM1158 metastatic melanoma cells that were treated for 1 hr with increasing doses of Elesclomol (20, 100, or 500 nM, data not shown). [9]. To determine whether Elesclomol treatment was linked to ROS produced during the process of melanin synthesis [14], we also treated the amelanotic metastatic melanoma cell line C32 with Elesclomol. C32 cells lack functional tyrosinase protein and therefore do not produce melanin in response to ultraviolet irradiation. As shown in Fig. 1, Elesclomol showed similar cytotoxicity in C32 cells as it did in the pigmented WM1158 cells, which indicates that non-melanosomal-containing organelles are important for Elesclomol-mediated increase in ROS.

### Elesclomol treatment inhibits melanoma cell proliferation

To determine whether Elesclomol treatment impairs the proliferation of melanoma cells we treated WM983-A and WM983-B melanoma cells with increasing doses of Elesclomol for 72 hr using a mitochondria based assay (MTT) as well as a cell proliferation assay that is based on the number of nuclei (CyQUANT). Table 1 shows that both assays yield comparable IC_50_ values. We then proceeded to assess the effect of Elesclomol treatment in cell proliferation in a larger panel of melanoma cell lines (WM1158, WM852, MV3, TPF10-741, TPF11-43, and Lu1205) as well as HEMs using the more broadly used MTT assay. The data presented in Table 1 document that compared with HEMs, proliferation of melanoma cells was impaired to a significantly greater extend. Proliferation of Vemurafenib-resistant metastatic melanoma cell lines TPF10-741 and TPF11-74 was also inhibited, albeit to a different extent.

**Table 1:** Proliferation of Elesclomol-treated HEMs and melanoma cells.

	20% O_2_ no CuCl_2_ (nM; 95CI)	20% O_2_ 5 µM CuCl_2_ (nM; 95CI)	0.1% O_2_, no CuCl_2_ (nM; 95CI)
HEMs	>500	216 (18–2,612)	
WM983-A	59 (5–782)	7 (1–38)	54 (12–247)
WM983-A_CyQUANT_	79 (23–267)	4 (2–12)	
WM983-B	47 (6–391)	26 (4–192)	63 (11–373)
WM983-B_CyQUANT_	90 (40–203)	2 (1–6)	
WM1158	31 (10–93)	11 (3–42)	34 (6–183)
WM852	14 (4–48)	6 (1–32)	30 (12–79)
MV3	110 (27–446)	5 (1–19)	164 (90–300)
TPF 10–741	>500	3 (1–11)	>500
TPF 11–43	59 (26–133)	23 (6–85)	116 (53–256)
Lu1205	6 (1–67)	1 (0.1–5)	37 (2–578)

Cells were grown for three days in 20% O_2_ in the absence or presence of CuCl_2_ (5 µM), or under hypoxic conditions (0.1% O_2_). Depicted are mean IC_50_ values for Elesclomol with 95% confidence intervals (95 CI) under each of the three conditions. ND - not determined.

To determine whether there might be a synergistic effect between Elesclomol and copper, we treated melanoma cells as well as HEMs with Elesclomol, or a combination of Elesclomol and copper chloride (5 µM). Elesclomol treatment combined with copper chloride led to greater inhibition of proliferation compared with Elesclomol treatment alone, and particularly in the case of the Vemurafenib-resistant melanoma cell line TPF10-741 (Table 1). To also investigate whether the effect of Elesclomol upon melanoma cell proliferation might be attenuated under hypoxic conditions, we treated WM983-A, WM983-B, WM1158, WM852, MV3, TPF10-741, TPF11-43, and Lu1205 melanoma cells with Elesclomol after the cells had been cultured for 72 hr in 0.1% oxygen. Compared with melanoma cells treated with Elesclomol under normoxic conditions, 0.1% oxygen had a variable effect on the different melanoma cell lines, although most melanoma cells were less sensitive to Elesclomol (S1).

### Elesclomol treatment of melanoma cells leads to suppression of mitochondria-associated proteins

To gain insights into possible changes in proteins associated with Elesclomol treatment, we performed a SILAC study of Elesclomol versus DMSO-treated melanoma cells. Mass spectrometry analysis of WM1158 cells treated with Elesclomol for 4 hr compared to WM1158 melanoma cells that had received only DMSO identified 733 proteins by at least two unique peptides; 1,066 proteins by at least two common peptides; and 166 proteins common to the two groups. Thus, the final proteomic analysis was based on (733+1,066)−252 = 1,633 identified proteins. In Elesclomol compared with DMSO-treated melanoma cells, 1,308 (79.8%) peptides were detected at lower levels. The proteomic data were then subjected to two statistical analyses. First, to IPA-Tox analysis [15] to identify cellular processes that are dysregulated in Elesclomol versus DMSO-treated melanoma cells. Using a 1.3-fold cutoff for changes in expression of a particular protein in Elesclomol versus DMSO-treated WM1158 cells, IPA-Tox analysis (Table 2) revealed that Elesclomol treatment significantly dysregulated proteins involved in ‘mitochondrial function’, ‘cholesterol biosynthesis’, ‘hypoxia-inducible factor signaling’, and as previously reported [9] ‘oxidative stress’. Presented in Table 3 are the proteins in each of the three top IPA-Tox categories, which showed that mitochondria-associated proteins were detected at lower levels in the Elesclomol-treated melanoma cells. The second statistical analysis we performed used ‘outlier’ analysis to identify the 5% of proteins that were dysregulated in the Elesclomol-treated melanoma cells. As shown in Table S1, 86 of these proteins were significantly dysregulated (58 upregulated; 28 downregulated) in melanoma cells that were treated with Elesclomol versus the drug vehicle. Similar to the IPA-Tox analysis, these data demonstrated dysregulation of proteins involved in various aspects of mitochondrial function (17% of the total number of dysregulated proteins). However, a significant number of proteins were not associated with mitochondrial function.

**Table 2:** Top 5 most significantly dysregulated pathways identified by SILAC and subsequent IPA-Tox analysis.

Pathways identified by IPA-Tox Analysis	Number of dysregulated proteins out of total number of proteins detected per pathway	p-value
Mitochondrial dysfunction	22/137	<0.001
Oxidative stress	13/57	<0.001
Cholesterol biosynthesis	6/16	<0.001
Increases transmembrane potential of mitochondria and mitochondrial membrane	9/50	<0.001
Hypoxia-inducible factor signaling	10/70	0.002

WM1158 cells treated for 4 hr with Elesclomol (500 nM) or only DMSO.

**Table 3:** Proteins from each of the Top 3 most significantly dysregulated pathways identified by SILAC and subsequent IPA-Tox analysis of WM1158 cells treated with Elesclomol (E) or the drug vehicle DMSO (V).

Symbol	Entrez Gene Name	UniProt	Peptide Count	Fold-reduction in E- versus V-treated cells
Mitochondrial Dysfunction				
CPT1A	Carnitine palmitoyltransferase 1A	P50416-1	6c	2.6
NDUFA7	NADH dehydrogenase (ubiquinone) 1 alpha subcomplex 7, 14.5 kDa (cmplx I)	O95182	9u	2.1
NDUFA2	NADH dehydrogenase (ubiquinone) 1 alpha subcomplex 2, 8 kDa (cmplx I)	O43678	4u	2.0
NDUFA10	NADH dehydrogenase (ubiquinone) 1 alpha subcomplex 10, 42 kDa (cmplx I)	O95299	2u+8c	1.9
PRDX5	Peroxiredoxin 5	P30044-1	26c	1.8
SDHA	Succinate dehydrogenase complex, subunit A, flavoprotein (cmplx II)	B3KYA5	5c	1.7
CYCS	Cytochrome c, somatic	P99999	4c	1.7
NDUFB11	NADH dehydrogenase (ubiquinone) 1 beta subcomplex 11, 17.3 kDa (cmplx I)	Q9NX14-1	4c	1.6
CASP3	Caspase 3, apoptosis-related cysteine peptidase	P42574	10c	1.6
NDUFA4	NADH dehydrogenase (ubiquinone) 1 alpha subcomplex 4, 9 kDa (cmplx I)	O00483	13u	1.6
NDUFS8	NADH dehydrogenase (ubiquinone) Fe-S protein 8, 23 kDa (cmplx I)	O00217	2c	1.6
SOD2	Superoxide dismutase 2, mitochondrial	P04179	13c	1.5
NDUFB4	NADH dehydrogenase (ubiquinone) 1 beta subcomplex 4,15 kDa (cmplx I)	O95168	5c	1.5
FIS1	Fission-1 (mitochondrial outer membrane) homolog (S. cerevisiae) (cmplx I)	Q9Y3D6	9u	1.5
UQCRC2	Ubiquinol-cytochrome c reductase core protein II (cmplx III)	P22695	32u	1.4
SDHB	Succinate dehydrogenase complex, subunit B, iron sulfur (cmplx II)	P21912	6u	1.4
NDUFB1	NADH dehydrogenase (ubiquinone) 1 beta subcomplex 1, 7 kDa (cmplx I)	A0AV68	4u	1.4
COX7A2L	Cytochrome c oxidase, subunit VIIa, polypeptide 2 like (cmplx IV)	O14548	4c	1.4
AIFM1	Apoptosis-inducing factor, mitochondrion-associated	O95831-1	10c	1.4
CAT	Catalase	P04040	24u	1.3
NDUFS5	NADH dehydrogenase (ubiquinone) Fe-S protein 5, 15 kDa (cmplx I)	O43920	6u	1.3
NDUFS7	NADH dehydrogenase (ubiquinone) Fe-S protein 7, 20 kDa (cmplx I)	O75251	3c	1.3
Oxidative stress				
SOD1	Superoxide dismutase 1, soluble	P00441	5u	2.1
STAT3	Signal transducer and activator of transcription 3 (acute-phase response factor)	P40763-1	4c	2.1
MAPK14	Mitogen-activated protein kinase 14	Q16539-1	2c	2.0
GSTM3	Glutathione S-transferase m3 (brain)	P21266	3u+10c	1.8
PRDX5	Peroxiredoxin 5	P30044-1	26c	1.8
PRDX6	Peroxiredoxin 6	P30041	57u+29c	1.7
ME1	Malic enzyme 1, NADP(+)-dependent, cytosolic	P48163	8u	1.6
GCLM	Glutamate-cysteine ligase, modifier subunit	P48507	2u	1.5
GSS	Glutathione synthetase	P48637	2u	1.5
SOD2	Superoxide dismutase 2, mitochondrial	P04179	13c	1.5
NQO1	NAD(P)H dehydrogenase, quinone 1	P15559	2u+22c	1.4
GPX1	Glutathione peroxidase 1	P07203	2u	1.4
CAT	Catalase	P04040	24u	1.3
Cholesterol Biosynthesis				
HMGCS1	3-hydroxy-3-methylglutaryl CoA synthase 1 (soluble)	Q01581	4u	2.2
LSS	Lanosterol synthase (2,3-oxidosqualene-lanosterol cyclase)	P48449	2u	2.0
PMVK	Phosphomevalonate kinase	Q15126	4u	1.7
FDFT1	Farnesyl-diphosphate farnesyltransferase 1	P37268	5c	1.7
FDPS	Farnesyl diphosphate synthase	B3KMW3	7c	1.6
ACAT2	Acetyl-CoA acetyltransferase	Q9BWD1	13u	1.3

u - proteins identified by unique peptides; c - proteins identified by common peptides.

Following these two statistical analyses, we used immunoblot blot analysis to validate the dysregulated levels of proteins in Elesclomol versus DMSO-treated melanoma cells. Using different melanoma cell lines, we determined the level of three different proteins. HO-1, because of the high number of tryptic peptides identified by the mass spectrometry analysis; TACO-1 because it was the most significantly suppressed protein in Elesclomol-treated melanoma cells (approximately 30-fold); and HIF-1α because the IPA-Tox analysis identified the HIF-1α pathway as one of the most dysregulated pathways following Elesclomol treatment (Table 2).

**Figure 2:**
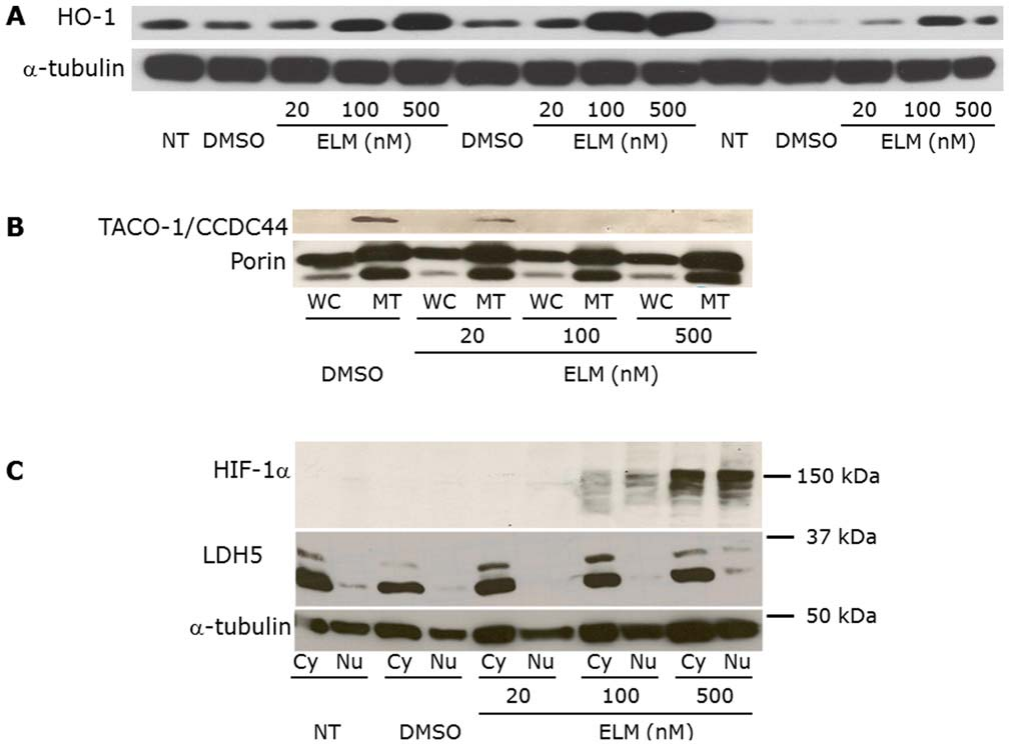
HO-1, TACO-1, and HIF-1α expression in Elesclomol-treated melanoma cells. (**A**) Whole-cell (WC), (**B**) mitochondrial and WC, and (**C**) nuclear (Nu) and cytoplasmic (Cy) lysates, prepared from WM1158 metastatic melanoma cells following treatment with increasing doses of Elesclomol (ELM). Controls were WM1158 melanoma cells that received the drug vehicle DMSO, or no treatment (no tx). The blots were probed with antibody to HO-1, TACO-1, HIF-1α, or α-tubulin, which served as loading control. LDH5 was used as a cytoplasmic protein control.

As shown in Fig. 2 and Fig. S1, HO-1 was upregulated in 3/3 melanoma cell lines (WM1158, WM983-B, and WM852); TACO-1 was downregulated in WM1158 melanoma cells and not detectable in two melanoma cell lines (Lu1205 and WM983-B) (data not shown); and HIF-1α was upregulated in 3/3 melanoma cell lines (WM1158, WM983-B, TPF10-741). To investigate the subcellular origin of HIF-1α and TACO-1, we performed immunoblot analysis comparing whole-cell lysates with mitochondrial extracts prepared from Elesclomol-treated melanoma cells, which showed that TACO-1 was decreased in mitochondrial extracts. HIF-1α was increased in both nuclear and cytoplasmic extracts of Elesclomol-treated melanoma cells.

### Elesclomol treatment of melanoma cells disrupts mitochondrial function

**Figure 3:**
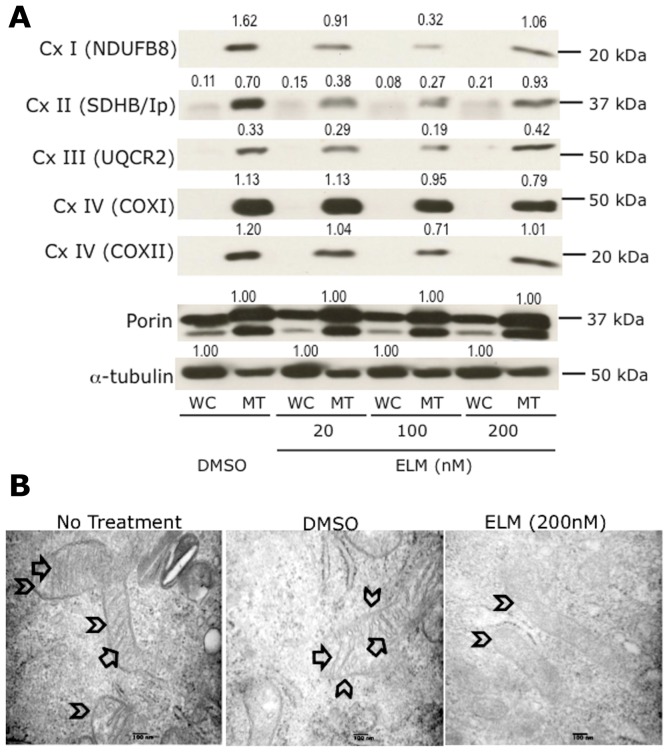
Impact of Elesclomol treatment on mitochondria. (**A**) Immunoblot analysis of whole-cell (WC) and mitochondrial (MT) lysates obtained from WM1158 cells that had been treated with Elesclomol (ELM) for 4 hr. The blots were probed with antibodies to various subunits that are part of complex I–IV of the mitochondrial respiratory chain. Signal intensity of the respective protein bands was normalized to α-tubulin (WC) and likewise, Porin (MT; two protein bands) using ImageJ imaging processing software. Abbreviations: NDUFB8-NADH dehydrogenase (ubiquinone) 1 beta subcomplex 8; SDHB/Ip-succinate dehydrogenase complex, subunit B, iron sulfur; UQCR2-ubiquinol-cytochrome c reductase core protein II; Sub 1-cytochrome c oxidase subunit 1; Cox II-cytochrome c oxidase subunit 2. (**B**) Electron micrograph images of mitochondria of WM1158 melanoma cells treated for 4 hr with 200 nM Elesclomol (ELM), or only drug vehicle (DMSO), or not treated (60,000× magnification). Arrowheads point to the outer mitochondrial membrane, and arrows to the inner mitochondrial membrane (cristae).

Since the proteomics data suggested that Elesclomol treatment induces changes in the abundance of mitochondrial respiratory chain complex subunits of I, II, III, and IV, combined with the fact that most of cellular ROS is produced by mitochondrial respiratory chain complexes [16], we investigated whether Elesclomol affects the expression or stability of the components of any of these complexes. Performing immunoblot analysis of whole-cell as well as mitochondrial extracts prepared from WM1158 and WM983-B melanoma cells that had been treated with increasing doses of Elesclomol (20, 100, or 500 nM) for 4 hr, we determined protein abundance of subunits representing each of the five mitochondrial respiratory chain complexes. Specifically, we used a MitoProfile total OXPHOS antibody cocktail, which contains antibodies to the following subunits that are labile when the corresponding complexes are not assembled or disrupted: NADH dehydrogenase (ubiquinone) 1 beta subcomplex 8 (NDUFB8; complex I); succinate dehydrogenase complex, subunit B, iron sulfur (SDHB/Ip; complex II); ubiquinol-cytochrome c reductase core protein II (UQCR2; complex III); cytochrome c oxidase subunit 2 (COXII; complex IV). In addition, we investigated the expression levels of the COXI (complex IV), the downstream effector of TACO-1, whose expression was suppressed in Elesclomol-treated melanoma cells. SDHB/Ip, UQCR2, and COXII were among the proteins identified by the SILAC analysis. For loading control of whole-cell lysates, we used an α-tubulin antibody. For loading control of mitochondrial lysates, we used an antibody to porin, a voltage-dependent anion-selective channel protein that resides in the outer mitochondrial membrane [17]. Our reason for selecting the latter was that the abundance of the two isoforms that were detected in the SILAC experiment (VDAC1 and VDAC3) was not different between Elesclomol and DMSO-treated melanoma cells (data not shown). Depicted in Fig. 3A is the dose-dependent suppression of all proteins at 20 nM and 100 nM of Elesclomol treatment. In contrast, at a dose of 500 nM of Elesclomol, a reduction was detected only in NDUFB8, COXI, and COXII. Similar dose-dependent downregulation of NDUFB8, SDHB/Ip, and UQCR2 was also observed in the case of WM983-B melanoma cells (data not shown). Thus, the results are in support of the SILAC data, which showed that Elesclomol treatment of melanoma cells disrupts mitochondrial components in the cells.

Further evidence that Elesclomol affects OXPHOS was obtained by an electron microscopy study of WM1158 melanoma cells treated with Elesclomol for 4 hr. Fig. 3B demonstrates that mitochondria from Elesclomol-treated cells do neither exhibit swelling nor disruption of the outer mitochondrial membrane. However, Elesclomol treatment did lead to morphologic changes in the inner mitochondrial membrane (loss of cristae), the site of the mitochondrial respiratory chain as well as numerous carrier proteins.

### Elesclomol suppresses OXPHOS without significant effects on glycolysis

Since the results of the studies presented above demonstrated that Elesclomol impairs the abundance of mitochondrial respiratory chain subunits in melanoma cells and alters the morphology of mitochondria (Fig. 3), we next investigated whether these changes would translate into quantitative changes in mitochondrial function. This was investigated using a Seahorse Flux analyzer, which measures oxygen consumption rate (OCR), a measure of OXPHOS, and extracellular acidification rate (ECAR), a measure of lactate production by glycolysis [18]–[20]. Using four different pharmacologic inhibitors, OCR and ECAR parameters in five melanoma cell lines (WM1158, WM983-B, Lu1205, TPF11-43, and TPF10-741) were measured in five different states. (1) basal state with addition of a metabolic inhibitor; (2) following addition of oligomycin to investigate whether the melanoma cells were metabolically flexible to increase glycolysis; (3) following addition of FFCP to investigate the respiratory reserve capacity, which is calculated by subtracting the OCR immediately prior to injection of oligomycin from the maximal OCR upon injection of FCCP [21]; (4) following addition of 2-DG to assess the flexibility of cells to switch from glycolysis to OXPHOS; and (5) following addition of rotenone to assess the contribution of OXPHOS to total respiration.

**Figure 4:**
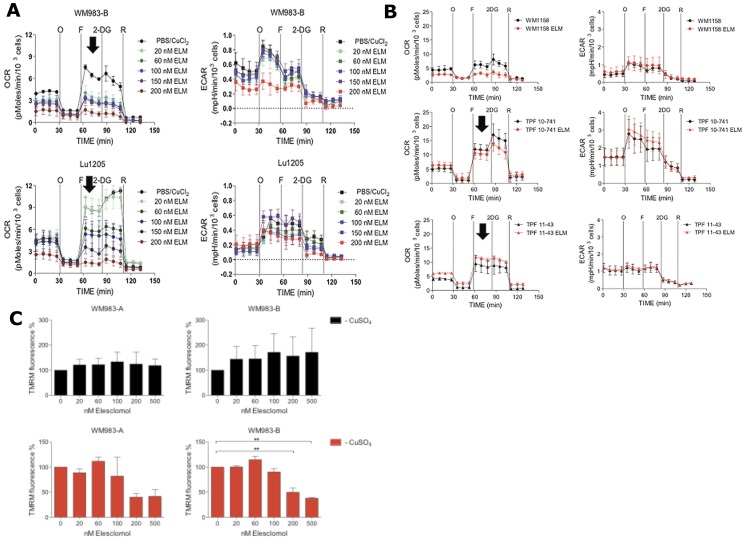
Bioenergetics analysis of melanoma cells. (**A**) Seahorse XF24 Flux analysis of Lu1205 and WM983-B metastatic melanoma cells treated for 2 hr with increasing doses of Elesclomol salt (ELM) (20, 60, 100, or 200 nM). After baseline OCR and ECAR determination, the cells were treated with oligomycin (O), FCCP (F), rotenone (R), or 2-deoxyglycose (2DG). Melanoma cells sensitive to Elesclomol, which had low reserve capacity and could not upregulate oxygen consumption in response to FCCP, are indicated by arrows. (**B**) Seahorse XF24 analysis of WM1158 cells and Vemurafenib-resistant melanoma cell lines (TPF10-741; TPF11-43) treated for 2 hr with 200 nM of Elesclomol salt (ELM) or only PBS. (C) Analysis of mitochondrial membrane potential in WM983-A and WM983-B using TMRM fluorescence following increasing doses of Elesclomol salt (20, 60, 100, 200, 500 nM) in the presence or absence of copper (5 µM).

To investigate whether Elesclomol has an immediate effect on cell metabolism and/or whether the presence of copper alone would alter metabolism, we treated the respective melanoma cells, prior to their analysis in the Seahorse Flux analyzer, for 2 hr with Elesclomol salt (200 nM) in culture medium containing copper (5 µM) or no copper. Only prior treatment with Elesclomol salt altered the bioenergetics of WM1158 and WM983-B melanoma cells (Fig. S2). Fig. 4A shows that a 2 hr treatment of WM983-B and Lu1205 melanoma cells with Elesclomol significantly impaired the reserve capacity for OXPHOS in a dose-dependent fashion to FCCP, which uncouples the proton gradient across the inner mitochondrial membrane. In addition, baseline OCR was suppressed in a dose-dependent fashion. As shown in Fig. 4B, suppression of an FCCP-induced increase in OCR was detected in WM1158 melanoma cells, but not in the Vemurafenib-resistant cell line TPF11-43 and the TPF10-741 cell line that has a high IC_50_ for Elesclomol (Table 1). With the exception of WM983-B melanoma cells treated at the highest dose of Elesclomol (200 nM), no changes in ECAR were detected in the various melanoma cell lines.

To explore whether Elesclomol suppresses OXPHOS via suppression of the mitochondrial membrane potential [22], we treated WM983-A and WM983-B melanoma cells with tetramethylrhodamine, methyl ester (TMRM), a lipophilic fluorescent cation probe that is accumulated by mitochondria in proportion to the electrical potential across the inner mitochondrial membrane (Δψ), in the presence of increasing concentration of Elesclomol salt alone or in combination with copper. Fig. 4C shows that Elesclomol treatment only in the presence of copper and at 200 and 500 nM dose levels decreased mitochondrial membrane potential at concentrations at 200 nM and 500 nM, well above the concentrations that inhibit cell growth and decrease OXPHOS. To assess whether the suppression of OXPHOS by Elesclomol leads to significant changes of intracellular ATP, we measured steady-state ATP levels, defined as ATP produced minus ATP consumed at any given time, in melanoma cells that were treated with Elesclomol salt versus no treatment. ATP levels remained constant for the duration of the experiment (Fig. S3). Maintenance of ATP levels is possible if melanoma cells decrease their metabolic requirements [23] in response to Elesclomol treatment, and either/or shifted their metabolism to increase glycolysis to maintain their ATP levels. Data presented in Fig. 4 would argue against the latter as ECAR levels, which are a direct indication of lactate being produced by glycolysis [23], [24] do not increase in response to Elesclomol.

### Inhibiting OXPHOS suppresses the cytotoxic effect of Elesclomol in melanoma cells

**Figure 5:**
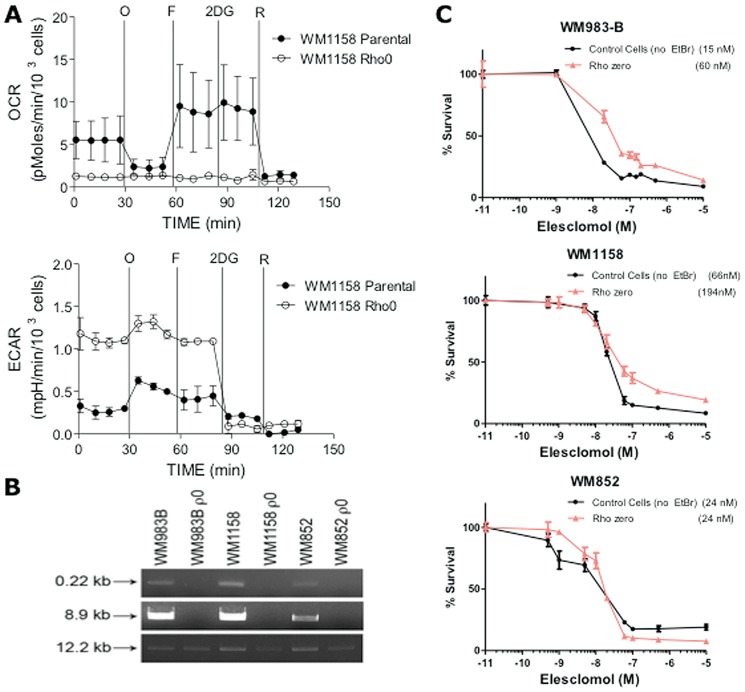
Analysis of ρ0 melanoma cells. (**A**) Equal amounts of DNA isolated from each cell line (parental; ρ0) were analyzed by qPCR for a small (0.22 kb) mitochondrial sequence (primer set 14,620/14,841), a large (8.9 kb) mitochondrial sequence (primer set 5,999/14,841), and a 12.2 kb DNA polymerase β primer set serving as a positive control for nuclear gene expression [25]. qPCR samples not containing DNA or primer sets served as negative controls. (**B**) Pharmacologic profile of OCR and ECAR of parental (solid circles) and ρ0 (open circles) WM1158 melanoma cell lines as determined by the Seahorse X24 analyzer. (**C**) Three**-**day MTT proliferation analysis of WM1158, WM983-B, and WM852 ρ0 and parental cells (no ethidium bromide, EtBr).

To obtain further evidence that Elesclomol mediates its action through the mitochondrial respiratory chain, we investigated whether a decreased reliance on OXPHOS attenuates the cytotoxic effect of Elesclomol on melanoma cells. Thus, we generated cells lacking mitochondrial DNA (ρ0 cells) from WM1158 and WM983-B melanoma cell lines, which had high baseline OCR, and from WM852 melanoma cells, which had low baseline OCR. Absence of mitochondrial DNA was confirmed by the quantitative polymerase chain reaction (qPCR). Loss of OXPHOS was determined by Seahorse XF24 analysis (Fig. 5A–B) as we have previously described [25]. To investigate whether ρ0 WM1158, WM983-B, and WM852 melanoma cells were more resistant to the cytotoxic effect of Elesclomol, a three-day standard MTT assay was performed. As depicted in Fig. 5, absence of mitochondrial DNA moderately increased the resistance of WM1158 and WM983-B melanoma cells to Elesclomol by approximately 3–4 fold. In contrast, it did not have an effect in WM852 cells that have low baseline OXPHOS. Interestingly, Elesclomol is equally toxic to all three cells at doses more than 100 nM, which suggests that Elesclomol may exert its cytotoxic effect via mechanisms other than direct effect on OXPHOS.

### Melanoma cells with reduced sensitivity to Elesclomol display increased glycolysis

**Figure 6:**
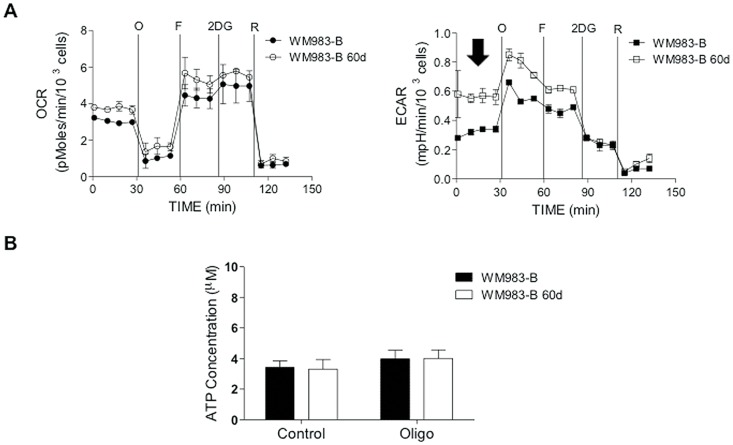
Selection of Elesclomol-resistant cells. (**A**) Following 60 days of every second day treatment of WM983-B cells with Elesclomol, a bioenergetics analysis was performed to measure the effects of continuous Elesclomol treatment upon OXPHOS and glycolysis. The arrow points to increased baseline ECAR in response to Elesclomol treatment for 60 days. (**B**) Measurement of steady-state ATP levels in WM983-B cells treated for 60 days with Elesclomol or only PBS.

To investigate whether continuous exposure of melanoma cells to Elesclomol would lead to metabolic changes, we cultured WM983-B cells in Elesclomol salt-containing medium that contained copper chloride (5 µM). Fresh medium with increasing Elesclomol concentrations was added every 2–3 days. WM983-B cells, grown in culture medium containing copper chloride (5 µM) served as the control. After 60 days of culture, WM983-B cells were able to proliferate in the presence of Elesclomol that was only twice as high as compared to the IC_50_ of the parental non-treated cells. Bioenergetics analysis of Elesclomol-treated and parental cells using Seahorse XF24 showed that the Elesclomol-resistant WM983-B cells did not have significant changes in OCR, but about two-fold higher ECAR (Fig. 6A). Analysis of steady-state ATP levels showed did not show significant differences between WM983-B melanoma cells treated with Elesclomol for 60 days and WM983-B cells not treated (Fig. 6B). Thus, it is likely that continuous exposure of melanoma cells to Elesclomol selects for cells that have higher rates of glycolytic ATP production.

### OXPHOS is an important metabolic pathway for melanoma cell lines

**Figure 7:**
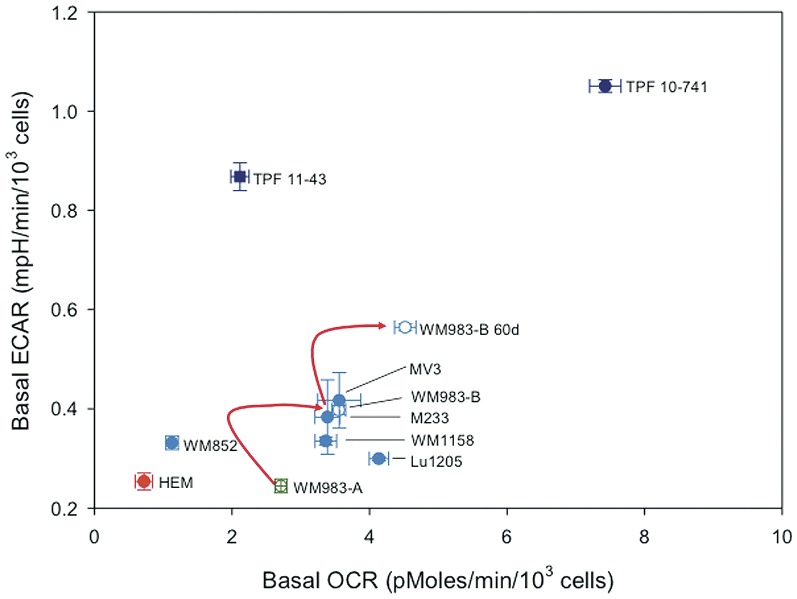
Basal OCR in relation to the ECAR in short-term cultures of human melanocytes, and primary and metastatic melanoma cell lines. Depicted are HEMs (red symbol), primary (green symbol) and metastatic melanoma cell lines (blue symbol), and two Vemurafenib-resistant melanoma cell lines (dark blue symbol). The two melanoma cell lines derived from tumors of a same patient are depicted by open circles.

The finding that Elesclomol, which has shown inconsistent evidence of a clinical benefit in patients with metastatic melanoma, affects components of the mitochondrial respiratory chain suggests that a particular group of patients with advanced melanoma rely on OXPHOS for energy production in addition to glycolysis [26]. To address this point, we performed bioenergetics analysis of HEMs and melanoma cell lines using the Seahorse XF24 analyzer. Fig. 7 shows the OCR in relation to the ECAR for HEMs and the melanoma cell lines. All of the melanoma cell lines had significantly higher OCR as compared to HEMs (*p*<0.05, one-way ANOVA followed by Dunnett's test). In addition, almost all of the metastatic melanoma cell lines analyzed had significantly higher ECAR compared with HEMs. Of note, the Vemurafenib-resistant MGP melanoma cell lines TPF10-741 and TPF11-43 exhibited significantly higher ECAR than the other metastatic melanoma cell lines. Also, the metastatic melanoma cell line WM983-B exhibited significantly higher rates of glycolysis and OXPHOS than the WM983-A cell line, which was isolated from the primary tumor of the same patient.

## Discussion

This study demonstrated that mitochondrial function exerts important functions relevant to melanoma cell survival and death, and that the drug Elesclomol targets a pertinent cellular function in melanoma. Specifically, we provide evidence that Elesclomol exerts its cytotoxic effect by inhibiting OXPHOS and mitochondrial respiration. Thus, Elesclomol should be considered along with the growing armament of compounds in various stages of drug development that target cancer metabolism [27], one of the key hallmarks of cancer [28].

Our Seahorse analysis showed that the melanoma cells, we analyzed, have about 6-fold higher levels of OXPHOS, as compared to HEMs and that Elesclomol decreases respiratory reserve capacity in mitochondria. Specifically, our data show that Elesclomol suppresses basal levels of OXPHOS and affects the ability of melanoma cells to upregulate OXPHOS in response to agents that either inhibit glycolysis, such as 2DG, or uncouple the mitochondrial proton gradient from ATP production, such as FCCP. The effect of Elesclomol on OXPHOS, at or below 100 nM concentrations, appears to be direct and not secondary to suppression of the mitochondrial membrane potential, a known regulator of OXPHOS [22]. This is an important finding because other non-mitochondrial effects of Elesclomol may account for its cytotoxic effect at least at high doses, as suggested by our SILAC analysis. Independent of the Seahorse analysis, the data we obtained via the SILAC study also showed that Elesclomol treatment decreases the abundance of distinct components from complex I of the mitochondrial respiratory chain, the ‘entry’ enzyme of OXPHOS that catalyzes the transfer of electrons from NADH to coenzyme Q (CoQ). The SILAC study also revealed that Elesclomol treatment decreased the abundance of several other components of the mitochondrial respiratory chain, including mitochondria-encoded proteins, such as TACO1 whose function per se is not associated with OXPHOS. Unlike in the case of the noticeable changes in OXPHOS, the changes we observed with respect to subunits in the mitochondrial respiratory chain were not substantial, but exhibited consistent downregulation. The latter is not surprising because the SILAC studies were performed during a time window of 4 hr. In addition, electron microscopy, showed that Elesclomol caused prominent loss of mitochondrial cristae, the site where components of the mitochondrial respiratory chain reside. We therefore conclude that Elesclomol does not exert its effect via a single molecular target within the mitochondrial respiratory chain, but through several molecules whose collective disruption leads to impairment of OXPHOS. Our results are in agreement with a recent study using yeast deletion mutants, which failed to identify a single molecule to account for the cytotoxic effect of elesclomol [29].

The other important aspect we experimentally addressed in the context of this study is whether Elesclomol retained its effect at a state when mitochondrial functions were impaired in the melanoma cells. For example, Elesclomol treatment during hypoxia, the latter of which is known to increase glycolytic enzymes and thus, shifts glucose utilization away from OXPHOS was less cytotoxic for most melanoma cell lines. These results can be explained in two different ways: First, cytochrome c oxidase activity remains saturated even at that low oxygen level [30]. Second, under stringent hypoxic conditions cells undergo metabolic reprogramming during which glutamine undergoes reductive carboxylation for lipid synthesis, allowing for cells to conserve glucose for production of ribose and other biosynthetic molecules [31], [32]. Another aspect we addressed was whether ρ0 melanoma cells that had been depleted of mitochondrial DNA remained sensitive to Elesclomol compared with parental cells containing mitochondrial DNA. Only the parental cells that exhibited higher rates of OXPHOS (WM1158 and WM983-B versus WM852) gave rise to ρ0 cells that were more resistant to Elesclomol. Ultimately, however, all three ρ0 melanoma cell lines were killed by relatively high doses of Elesclomol. Our working hypothesis is that while these ρ0 cells no longer perform OXPHOS, these cells in order to survive, maintain a proton gradient across the inner mitochondrial membrane by running complex V in reverse consuming ATP [33]. Our data indicated that Elesclomol at high doses (≥200 nM) decreases mitochondrial membrane potential and therefore can disrupt the inner mitochondrial membrane proton gradient, which then lead to cell death. This high dose effect of Elesclomol would be expected to be independent of the presence of oxygen, as in the case of our hypoxic experiments, and in the absence of functional OXPHOS, as in the case of our ρ0 cell experiments.

In contrast with the Warburg hypothesis, which states that a large proportion of ATP is produced by tumor cells via glucose metabolism with concomitantly decreased ATP produced by oxidation of mitochondrial substrates caused by mitochondrial defects [34], our findings across multiple cell lines show that melanoma cells exhibit substantially higher rates of OXPHOS than HEMs. Our findings regarding the role of OXPHOS in advanced melanoma are in line with a previous report [35], which documented that compared with other solid tumor xenografts, human melanoma xenografts have one of the highest rates of oxygen consumption, a surrogate marker of OXPHOS. In addition, it has been reported that ρ0 melanoma cells do not form xenografts [36], and that non-glycolytic metabolic sources, such as the Krebs cycle, are more prominent in melanoma cells compared with melanocytes [37].

The other important and novel finding of our study is that even at nanomolar concentrations, Elesclomol had a cytotoxic effect on melanoma cells whereas HEMs were largely resistant. Since the HEMs exhibited low rates of OXPHOS and thus generate lower levels of endogenous ROS, it is possible that cells are less sensitive to Elesclomol inhibition of OXPHOS, and their antioxidant capacity is capable of quenching Elesclomol-induced ROS. In contrast, since we found that melanoma cells exhibit high rates of OXPHOS, it is likely that they have high levels of endogenous ROS and therefore, their antioxidant reserve is not sufficient to defend against the additional ROS burden induced by Elesclomol. Thus, further induction of oxidative stress by Elesclomol likely exceeds the antioxidant capacity of melanoma cells, leading to cell death [9], [38].

The specific effect of Elesclomol on the mitochondrial respiratory chain and OXPHOS suggests possible mechanisms of resistance. TPF10-741, a melanoma cell line that is resistant to Vemurafenib and as we show, Elesclomol, exhibited the highest levels of ECAR. However, this trend is not universal because the Vemurafenib-resistant melanoma cell lines TPF11-43 was relatively sensitive to Elesclomol at median nanomolar concentrations. Regarding secondary resistance to Elesclomol reflected by the finding that we could not select for melanoma cells with resistance to Elesclomol at an IC_50_ value higher than 500 nM, we found that 60 days continuous exposure of WM983-B cells to Elesclomol selected for cells with significantly higher glycolysis. The molecular mechanisms behind this phenomenon may involve, among others, HIF-1α upregulation. A pertinent molecule for melanoma [39], HIF-1α is involved in suppressing OXPHOS, upregulates the metabolic program involved in glycolysis, and shows an association with overall worse prognosis in multiple cancers [40].

It is known that melanoma patients with either high serum LDH or high expression of LDH5, the LDH isoenzyme involved in the biochemical conversion of pyruvate to lactate, have poor prognosis [5], [13]. Thus, our findings regarding Elesclomol have important clinical implications in relation to a large failed phase III trial of this drug. The specific effect of Elesclomol upon cells utilizing OXPHOS, but not glycolysis, may explain why Elesclomol has a potential clinical benefit only in patients with metastatic melanoma and normal serum LDH, a patient subgroup with better prognosis [13], whereas in patients with high serum LDH, Elesclomol treatment may have an adverse effect upon overall survival [12]. Given our data, it is possible that the metabolism of melanoma cells in these patients is more dependent upon OXPHOS for energy production as opposed to glycolysis, a hypothesis we are currently testing.

Lastly, our SILAC, immunoblot, and electron microscopy data along with the finding that we could not generate a ‘bona fide’ Elesclomol-resistant melanoma cell line are in agreement with the result from a recent study, which indicate that multiple protein targets account for the cytotoxic effect of Elesclomol [29]. This suggests that only conditions that shift metabolic balance towards glycolysis and not individual changes in particular proteins of the mitochondrial respiratory chain account for the resistance to Elesclomol.

## Materials and Methods

### Cell cultures

Human epidermal melanocytes (HEMs) were purchased from Cell Applications (San Diego, CA) and propagated as per the manufacturer's recommendation. Human melanoma cell lines (WM1158, WM852, WM983-A, WM983-B, Lu1205, C32) were purchased from the Coriell Institute for Medical Research (Camden, NJ) or the American Type Culture Collection (Manassas, VA). MV3 melanoma cells were established by Dr. D.J Ruiter (University Hospital Nijmegen) and obtained from Dr. S. Ferrone (University of Pittsburgh) [41], and M233 melanoma cells [42] were established and provided by Dr. A. Ribas (UCLA). The human cell lines TPF10-741 and TPF11-43 were established at our institution (JMK, CS, YY) under the University of Pittsburgh Cancer Institute (UPCI) tissue banking protocol UPCI96-099 that allows acquisition of fresh tumor tissue from patients with metastatic melanoma who have signed written informed consent. Both cell lines were established from subcutaneous metastases of two patients who developed secondary resistance to Vemurafenib after an initial partial response in a phase II trial of Vemurafenib in metastatic melanoma (BRIM-2) [43]. Immunoblot analysis of whole-cell lysates prepared from TPF10-741 and TPF11-43 cells and probed with antibodies to S100 antigen, Melan-A, tyrosinase, and MAGE-A served to confirm that they were melanoma cells. Melanoma cell lines were made deficient of mitochondrial DNA (rho zero, ρ0) as previously described [25]. For experiments performed under hypoxic conditions, a hypoxic chamber (Billups-Rothenberg, Inc., Del Mar, CA) and premixed gas (0.1% O_2_, 5% CO_2_ balanced with N_2_) were used as previously described [44].

### Drugs and antibodies

Two different formulations of Elesclomol, provided by Synta Pharmaceuticals, Inc. (Lexington, MA) were used: Elesclomol soluble in DMSO, and Elesclomol salt, soluble in phosphate buffered saline (PBS). Copper (CuCl_2_) was purchased from Sigma-Aldrich (St. Louis, MO). Antibodies were Heme Oxygenase-1 (HO-1) (rabbit anti-human monoclonal, Epitomics, Burlingame, CA), hypoxia inducible factor-1alpha (HIF-1α) (mouse anti-human monoclonal, BD Biosciences, San Diego, CA), α-tubulin (rabbit anti-human monoclonal, Cell Signaling, Danvers, MA), Mitoprofile total OXPHOS human Western blot antibody cocktail (MitoSciences, Eugene, OR), OXPHOS complex IV subunit I (mouse monoclonal anti-human, Invitrogen, Carlsbad, CA), and Porin (mouse anti-human monoclonal, MitoSciences). Translational activator of cytochrome c oxidase subunit-1 (TACO-1) (rabbit anti-human polyclonal antibody) was provided by Dr. Eric Shoubridge (McGill University).

### Cell proliferation analysis

Melanoma cells were seeded, in triplicate, into 96-well tissue culture plates. Following overnight incubation, the medium was replenished with fresh medium containing increasing concentrations of Elesclomol or only DMSO. Seventy-two hr following drug treatment, cell viability was determined using the the CyQUANT Cell Proliferation Assay and the Vybrant MTT Cell Proliferation Assay Kit (both from Invitrogen). Using CalcuSyn Version 2.1 (Biosoft, Cambridge, UK), IC_50_ values were determined using the Chou's median-effect equation, correlating dose and effect via the following formula f_a_/f_u_ = (D/D_m_)m, with *D* being dose of the drug; *D_m_*, median-effect dose signifying the potency; *f_a_* fraction affected by the dose; *f_u_* fraction unaffected (i.e. f_u_ = 1−f_a_), and *m* an exponent signifying the sigmoidicity (shape) of the dose-effect curve.

### SILAC analysis

As previously described [45], melanoma cells were grown in medium supplemented with ‘heavy’ arginine (^13^C_6_^15^N_4_ L-Arg) and lysine (^13^C_6_ L-Lys), or corresponding ‘light’ amino acids. Cells grown in ‘heavy’ media were treated with Elesclomol, and cells grown in ‘light’ amino acid-containing medium were treated with the drug vehicle, DMSO. Whole-cell lysates, prepared from Elesclomol or DMSO-treated cells, were mixed at a ratio of 1∶1, followed by sodium dodecyl sulfate polyacrylamide gel electrophoresis (SDS-PAGE). Peptides were analyzed in duplicates by liquid chromatography Mass Spectrometry (LC-MS,) and queried using a human proteome database [Uniprot, version (10/2008)]. For each protein, the SILAC ratio (Elesclomol-treated)/(Drug vehicle-treated) was normalized to the mean ratio and standard deviation for the entire data set. Proteins below two standard deviations from the mean (outside of a 95% confidence interval; two-tailed distribution) were discerned as significantly upregulated or downregulated. In this transformed distribution, two tails equal to 5% exist, i.e. 2.5% for the majority of upregulated proteins and 2.5% for the majority of downregulated proteins. For determination of cellular processes associated with Elesclomol-treated melanoma cells, identified proteins with fold changes of ≥1.3 were interrogated in Ingenuity Pathway Analysis Toxicology (IPA-Tox) (Ingenuity Systems).

### Immunoblot blot analysis and electron microscopy

Whole-cell lysates were prepared using 10× lysis buffer [46]. Nuclear and cytoplasmic extracts were isolated using the NE-PER nuclear and cytoplasmic extraction kit (Thermo Scientific). Mitochondria isolated from melanoma cells using a mitochondrial isolation kit (Mitosciences) were stored at −80°C until further use. Mitochondrial proteins were extracted from snap-frozen mitochondria by adding 0.2% SDS. Protein lysates (30 µg/sample), prepared from whole cells or subcellular fractions, were separated on 10% SDS-PAGE, transferred onto nitrocellulose membrane, and probed with primary antibody followed by incubation with a horseradish peroxidase-conjugated secondary antibody (Cell Signaling), and chemiluminescent HRP Substrate (Millipore). For electron microscopy studies, melanoma cells were treated with Elesclomol (200 nM for 4 hr), fixed with 2% glutaraldehyde for 30 min at room temperature, and stored at 4°C until further analysis. Samples were analyzed by electron microscopy as previously described [47].

### Seahorse XF24 Flux Analyzer

The Seahorse XF24 Flux analyzer (Seahorse Biosciences, Billerica, MA) was used to determine the metabolic profiles of melanoma cells [25]. 4×10^4^ cells/well were seeded into Seahorse XF24 microplates and incubated at 37°C for approximately 24 hr. Thereafter, the cells were treated for 2 hr with different doses of Elesclomol salt in combination with copper chloride (5 µM), or likewise with different doses of Elesclomol in the absence of copper. Basal oxygen consumption rate (OCAR) and extracellular acidification rate (ECAR) were measured in the Seahorse XF24 Flux analyzer. Additional measurements were performed after injection of four compounds affecting bioenergetics: (oligomycin (1 µM) (Sigma-Aldrich), carbonyl cyanide 4-trifluoromethoxy-phenylhydrazone (FCCP) (300 nM) (Sigma-Aldrich), 2-deoxyglucose (2-DG), (100 mM) (Sigma-Aldrich), and rotenone (1 µM) (Sigma-Aldrich). Upon completion of the Seahorse XF24 Flux analysis, cells were trypsinized, counted, and the results were normalized per 10^3^ cells. Statistical analysis was performed using one-way analysis of variance (ANOVA), followed by the Dunnett's test.

### Mitochondrial membrane potential analysis

Melanoma cells were seeded in duplicates, into 96-well tissue culture plates (1.5×10^4^ cells/well). Following overnight incubation, fresh medium (50 µL) containing increasing concentrations of Elesclomol salt in combination with copper chloride (5 µM), or likewise with different doses of Elesclomol in the absence of copper, or the drug vehicle (PBS) was added to cells. Six hours following drug treatment, cells were rinsed twice with DMEM/L15 medium without phenol red and incubated for 15 min at 37°C with 10 µM tetramethylrhodamine, methyl ester (TMRM, Molecular Probes). Fluorescent measurements were obtained on a Biotek Synergy 2 plate reader (Winooski, VT). Statistical analysis was performed using one-way analysis of variance (ANOVA), followed by the Tukey's multiple comparison test.

### ATP measurements

Steady-state ATP levels were measured using a luminescence ATP detection assay (ATPlite PerkinElmer Inc., Waltham, MA). 4×10^4^ cells/well were grown overnight in 96-well black plates and treated separately with the indicated compounds for 45 min, followed by cell lysis using 50 µl of cell lysis solution, and incubation for 5 min at 300 rpm. Thereafter, substrate solution (50 µl) was added, and the microplates were incubated for 5 min at 270 rpm. The plates were kept in the dark for 10 min, and luminescence was measured using a Biotek Synergy 2 plate reader (Winooski, VT). Statistical analysis was performed using one-way analysis of variance (ANOVA).

## Supporting Information

Figure S1 **HIF-1α and HO-1 analysis of Elesclomol-treated melanoma cells.** (**A**) HIF-1α immunoblot analysis of whole-cell lysates prepared from WM983-B and TPF10-741 melanoma cells treated for 6 hr with increasing doses of Elesclomol (ELM) (20, 100, or 500 nM). (**B**) HO-1 immunoblot analysis of whole-cell lysates prepared from WM983-B and TPF10-741 melanoma cells treated with increasing doses of Elesclomol (ELM) (20, 100, or 500 nM) for 4, 8, or 24 hr. Cells not treated or treated with drug vehicle, DMSO, served as controls.
(TIFF)

Figure S2 **Bioenergetics analysis of WM1158 and WM983-B melanoma cells.** Cells treated with 200 nM of Elesclomol salt in the presence of 5 µM copper (ELM), PBS/CuCl_2_ (5 µM), or only PBS (control). Elesclomol was administered either via a 2 hr incubation (ELM incubated), or by injection from port A of the Seahorse XF24 Flux analyzer (ELM injected). After determination of baseline OCR and ECAR, the cells were treated with oligomycin (O), FCCP (F), and rotenone (R).
(TIFF)

Figure S3 **Steady-state ATP levels in melanoma cells treated with Elesclomol salt.** Melanoma cells were treated for 2 hr with 200 nM of Elesclomol salt (ELM) or only PBS (NT). Thereafter, the cells were treated for 45 min with 1 µM of oligomycin (oligo) or only DMSO (control).
(TIFF)

Table S1 **Dysregulated proteins identified by outlier analysis of the SILAC data of WM1158 melanoma cells treated with Elesclomol (E) versus the drug vehicle DMSO (V).** Results are presented as E/V ratio. Highlighted (boldface) are proteins associated with mitochondrial functions. Abbreviations: u - proteins identified by unique peptides; c - proteins identified by common peptides.
(DOCX)
