# Supplementary figures and images for: Mitochondrial Respiration - An Important Therapeutic Target in Melanoma
Source: PLoS One. 2012 Aug 17;7(8):e40690. doi: 10.1371/journal.pone.0040690 (PMC3422349; doi:10.1371/journal.pone.0040690)

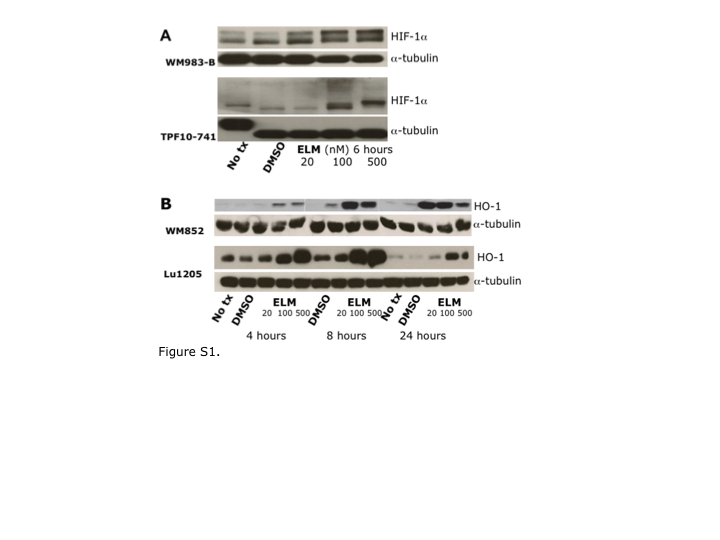

Supplement: Figure S1 — HIF-1α and HO-1 analysis of Elesclomol-treated melanoma cells. (A) HIF-1α immunoblot analysis of whole-cell lysates prepared from WM983-B and TPF10-741 melanoma cells treated for 6 hr with increasing doses of Elesclomol (ELM) (20, 100, or 500 nM). (B) HO-1 immunoblot analysis of whole-cell lysates prepared from WM983-B and TPF10-741 melanoma cells treated with increasing doses of Elesclomol (ELM) (20, 100, or 500 nM) for 4, 8, or 24 hr. Cells not treated or treated with drug vehicle, DMSO, served as controls. (TIFF) [file pone.0040690.s001.tiff]

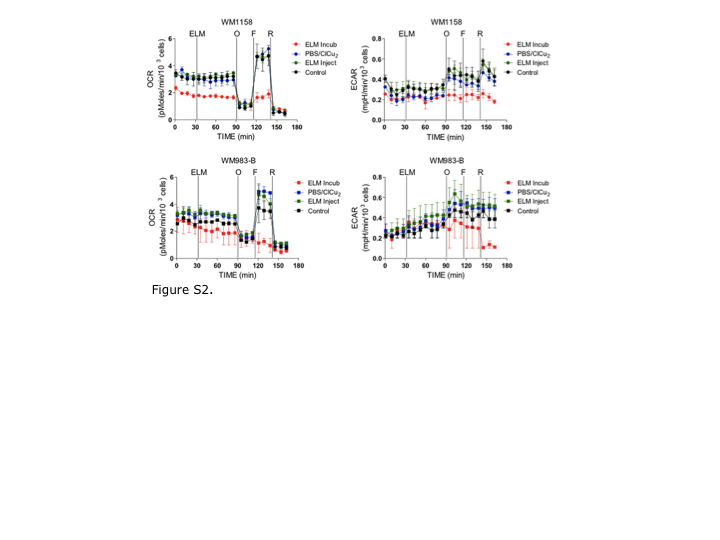

Supplement: Figure S2 — Bioenergetics analysis of WM1158 and WM983-B melanoma cells. Cells treated with 200 nM of Elesclomol salt in the presence of 5 µM copper (ELM), PBS/CuCl2 (5 µM), or only PBS (control). Elesclomol was administered either via a 2 hr incubation (ELM incubated), or by injection from port A of the Seahorse XF24 Flux analyzer (ELM injected). After determination of baseline OCR and ECAR, the cells were treated with oligomycin (O), FCCP (F), and rotenone (R). (TIFF) [file pone.0040690.s002.tiff]

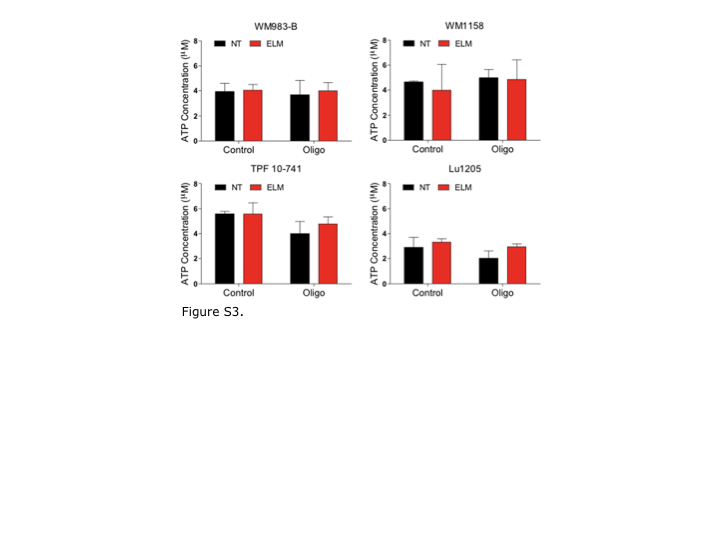

Supplement: Figure S3 — Steady-state ATP levels in melanoma cells treated with Elesclomol salt. Melanoma cells were treated for 2 hr with 200 nM of Elesclomol salt (ELM) or only PBS (NT). Thereafter, the cells were treated for 45 min with 1 µM of oligomycin (oligo) or only DMSO (control). (TIFF) [file pone.0040690.s003.tiff]
