# Supplementary material for: Mitochondrial Respiration - An Important Therapeutic Target in Melanoma
Source: PLoS One. 2012 Aug 17;7(8):e40690. doi: 10.1371/journal.pone.0040690 (PMC3422349; doi:10.1371/journal.pone.0040690)
Supplement: Table S1 — Dysregulated proteins identified by outlier analysis of the SILAC data of WM1158 melanoma cells treated with Elesclomol (E) versus the drug vehicle DMSO (V). Results are presented as E/V ratio. Highlighted (boldface) are proteins associated with mitochondrial functions. Abbreviations: u - proteins identified by unique peptides; c - proteins identified by common peptides. (DOCX) [file pone.0040690.s004.docx]

| **Protein** | **Function** | **UniProt No** | **Peptide Count** | **Ratio E/V** |
| --- | --- | --- | --- | --- |
| Beta-hexosaminidase subunit β | Degrades GM2 gangliosides | P07686 | 3 | 1.78 |
| **Thioredoxin domain-containing 13** | Oxidoreductase involved in disulfide bond formation | Q9H1E5 | 2 | 1.74 |
| Proteolipid protein 2 | Facilitates melanoma metastases | Q04941 | 2 | 1.74 |
| Dolichyldiphosphatase 1 | Required for N-glycosylation | Q86YN1 | 2 | 1.68 |
| Ser/Thr Protein phosphatase 6 | Involved in cell cycle progression in response to IL-2 | O00743 | 4 | 1.67 |
| Signal recognition particle 9 kDa | Role in targeting secretory proteins to rough ER | P49458 | 6 | 1.61 |
| **ATP synthase subunit δ** | Subunit of mitochondrial ATPase | P30049 | 6 | 1.58 |
| Histone cluster 1, H3A | Core component of the nucleosome | P68431 | 14 | 1.56 |
| Histone cluster 3, H3 | Core component of the nucleosome | Q16695 | 4 | 1.53 |
| **Succinyl-CoA ligase subunit α** | Succinyl CoA+ADP(GDP)🡪Succinate+ATP(GTP) | P53597 | 6 | 1.51 |
| DDRGK1 | ER protein with role in ER-reorganization | Q96HY6 | 3 | 1.51 |
| **Acyl-CoA thioesterase 13** | Catalyzes hydrolysis of acyl-CoA to FFAs and coenzyme A (CoASH) | Q9NPJ3 | 5 | 1.47 |
| WD repeat domain 61 | Role in transcriptional regulation | Q9GZS3 | 4 | 1.44 |
| Chromosome 15 ORF 48 | Low or absent in esophageal squamous cell carcinoma | Q9C002 | 2 | 1.44 |
| Heme oxygenase 1 | Cleaves heme to form biliverdin | P09601 | 11 | 1.40 |
| U3 snRNA-interacting protein 2 | Participates in the processing and modification of pre-ribosomal RNA | O43818 | 6 | 1.40 |
| **28S ribosomal protein S7** | Component of the mitochondrial ribosome small subunit (28S) | Q9Y2R9 | 5 | 1.39 |
| Structural maintenance of chromosomes protein 1A | Component of the cohesion complex that is involved in the proper cohesion of sister chromatids; is phosphorylated by ATM | Q14683 | 4 | 1.38 |
| 40S ribosomal protein S8 | Component of the 40S subunit of cytoplasmic ribosomes | P62241 | 2 | 1.37 |
| Isoform 1 of Apolipoprotein O | Promotes cholesterol efflux; involved in protective mechanisms against lipid accumulation | Q9BUR5 | 3 | 1.37 |
| Long Isoform of Ki-67 Antigen | Nuclear protein expressed during late G1, S, G2, M phases of the cell cycle; it may be necessary for cell proliferation | P46013 | 7 | 1.32 |
| Probable saccharopine dehydrogenase | Oxidoreductase that is involved in lysine degradation | Q8NBX0 | 2 | 1.32 |
| Isoform 1 of catenin α1 | Associates with the cytoplasmic domain of various cadherins; may regulate actin filament assembly and actin branching | P35221 | 2 | 1.29 |
| Isoform 1 of phosphatidylinositol glycan anchor biosynthesis class U protein | Component of the glycosylphosphatidylinositol transamidase complex | Q9H490 | 2 | 1.28 |
| HEAT repeat-containing protein 1 | Phosphorylated by ATM/ATR and is involved in the nucleolar processing of pre-18S rRNA | Q9H583 | 16 | 1.26 |
| Ras-related protein Rab-11B | GTPase that modulates endosomal trafficking | Q15907 | 4 | 1.26 |
| Isoform 1 of Zinc finger protein castor homolog 1 | Probable transcription factor, putative survival related zinc finger protein | Q86V15 | 2 | 1.24 |
| Tetraspanin-24 | Cell surface glycoprotein involved in cell adhesion/motility, integrin trafficking/function | P48509 | 5 | 1.24 |
| **Transmembrane protein 14C** | Required for heme biosynthesis (by similarity) | Q9P0S9 | 8 | 1.24 |
| **Cyclohydrolase** | One carbon metabolism | P13995 | 4 | 1.24 |
| Proline-rich and coiled-coil-containing protein 2C | Unknown | Q9Y520 | 3 | 1.24 |
| Stathmin | Destabilizes microtubules | P16949 | 2 | 1.24 |
| Superkiller viralicidic activity 2-like 2 | Associated with the RNA exosome complex and involved in the 3' processing of the 7S pre-RNA to the mature 5.8S rRNA | P42285 | 7 | 1.24 |
| **39S ribosomal protein L32** | Component of the mitochondrial ribosome large subunit (39S) | Q9BYC8 | 3 | 1.23 |
| 60S ribosomal protein L26-like 1 | Unknown | Q9UNX3 | 5 | 1.23 |
| Isoform 1 of splicing factor, Arg/Ser-rich 10 | Sequence-specific RNA-binding protein involved in pre-mRNA splicing | P62995 | 7 | 1.23 |
| Isoform 1 of ER aminopeptidase 1 | Aminopeptidase that plays a central role in peptide trimming required for presentation on MHC class I molecules | Q9NZ08 | 4 | .1.22 |
| Histone H2B type 1-D | Core component of nucleosome, a histone octamer (H2A-H2B-H3-H4) | P58876 | 22 | 1.22 |
| Histone H2A type 1-D | Core component of nucleosome, a histone octamer (H2A-H2B-H3-H4) | P20671 | 8 | 1.21 |
| Cytoskeleton-associated protein 5 | Microtubule-associated protein that binds to hnRNP | Q14008 | 7 | 1.21 |
| Isoform 1 of translocon-associated protein subunit α | Member of a complex that binds calcium to the ER membrane and regulates retention of ER resident proteins | P43307 | 5 | 1.21 |
| Histone cluster 2, H3, pseudogene 2 | Unknown | Q5TEC6 | 4 | 1.20 |
| **NAD-dependent malic enzyme** | MM enzyme involved in the conversion of malate to pyruvate | P23368 | 2 | 1.20 |
| **Mitochondrial import inner membrane translocase subunit Tim13** | Mitochondrial intermembrane chaperone that participates in the import and insertion of some multi-pass transmembrane proteins into the mitochondrial inner membrane | Q9Y5L4 | 3 | 1.20 |
| Inositol 1,4,5-trisphosphate receptor type 3 | Ligand-gated ion channel that is activated by cytosolic Ca^2+^ and 1,4,5-triphoshate | Q14573 | 5 | 1.20 |
| **28S ribosomal protein S18b** | Component of the mitochondrial ribosome small subunit (28S) | Q9Y676 | 3 | 1.98 |
| Zinc finger protein with KRAB and SCAN domains 5 | Unknown | Q9Y2L8 | 3 | 1.19 |
| Ferritin light polypeptide variant (Fragment) | Light subunit of ferritin, a protein involved in iron storage in a soluble and nontoxic state; variation in ferritin subunit composition may affect rates of iron uptake and release in different tissues | B1Q3B4 | 2 | 1.19 |
| Condensin complex subunit 3 | Regulatory subunit of the condensin complex that is required for conversion of interphase chromatin into mitotic-like chromosomes | Q9BPX3 | 5 | 1.19 |
| Synapse-associated protein 1 | PZD-domain protein predominantly localized in the postsynaptic density | Q96A49 | 2 | 1.18 |
| Isoform 1 of Collagen alpha-3(VI) chain | One of the three chains of Collagen VI, a cell-binding protein | P12111 | 40 | 1.18 |
| Probable E3 ubiquitin-protein ligase TRIP12 | Component of PA700, an ATP-dependent multisubunit protein that activates the proteolytic activities of the 20S proteasome | Q14669 | 9 | 1.18 |
| Sialic acid synthase | Enzyme involved in the sialic acid biosynthesis | Q9NR45 | 2 | 1.18 |
| Histone H2B type 1-B | Core component of nucleosome, a histone octamer (H2A-H2B-H3-H4) | P33778 | 57 | 1.18 |
| Isoform 2 of RNA-binding protein Raly | Protein that may be involved in pre-mRNA splicing. | Q9UKM9 | 3 | 1.17 |
| Probable RNA-binding protein 25 | Protein that regulates alternative pre-mRNA splicing and is involved in apoptotic cell death through regulation of BCL2L1 isoform expression | P49756 | 3 | 1.17 |
| FACT complex subunit SSRP1 | Component of the FACT complex, a general chromatin factor that reorganizes nucleosomes and is involved in processes that require DNA as a template (mRNA elongation, DNA replicaton/repair) | Q08945 | 4 | 1.16 |
| Isoform 1 of DNA-dependent protein kinase catalytic subunit | Ser/Thr-protein kinase that acts as a molecular sensor for DNA damage and is involved in DNA non-homologous end joining | P78527 | 129 | 1.15 |
| Isoform 1 of 2',5'-phosphodiesterase 12 | Enzyme that cleaves 2',5'-phosphodiester bond linking adenosines of the 5'-triphosphorylated oligoadenylates and triphosphorylated oligoadenylates (2-5A system) | Q6L8Q7 | 2 | 0.37 |
| Protein SEC13 homolog | Component of the nuclear pore complex and the COPII coat | P55735 | 6 | 0.37 |
| Long isoform of 60 kDa SS-A/Ro RNP | RNA-binding protein that binds to and stabilizes several small cytoplasmic RNA molecules (Y RNAs) | P10155 | 2 | 0.36 |
| Long isoform of TATA-binding protein-associated factor 2N | RNA and ssDNA-binding protein that may play specific roles during transcription initiation at distinct promoters | Q92804 | 2 | 0.36 |
| Acyltransferase-like 1 | Possess ac(et)yltransferase activity | Q7L5N7 | 3 | 0.34 |
| Isoform 1 of RNA-binding protein with serine-rich domain 1 | Involved in the formation of complex A of the spliceosome | Q15287 | 2 | 0.34 |
| **Glutamate/H(+) symporter 1** | Transports glutamate across inner mitochondrial membrane | Q9H936 | 2 | 0.33 |
| Core-binding factor subunit β | Regulatory subunit of PEBP2/CBF transcription factor family | Q13951 | 2 | 0.32 |
| Lysyl oxidase homolog 2 | Catalyzes first step in collagen/elastin crosslinks | Q9Y4K0 | 2 | 0.30 |
| G protein subunit β2 | Required for GDPase activity of G proteins | P62879 | 9 | 0.30 |
| Chloride intracellular channel 2 | Inserts into membranes under oxidizing conditions | O15247 | 3 | 0.29 |
| **Long-chain-fatty-acid--CoA synthetase 4** | Activates long chain fatty acids for metabolism (synthesis or degradation) | O60488 | 4 | 0.29 |
| α-Ketoglutarate-dependent dioxygenase FTO | Repairs alkylated nucleic acids by oxidative demethylation | Q9C0B1 | 2 | 0.28 |
| Transducin β chain 1 | Required for GDPase activity of G proteins | P62873 | 13 | 0.27 |
| **Inorganic pyrophosphatase 2** | Enzyme that breaks down diphosphate into two phosphates | Q9H2U2 | 3 | 0.26 |
| Isoform A of trypsin-3 | Degrades trypsin inhibitors | P35030 | 2 | 0.26 |
| Cell division cycle protein 123 homolog | Required for S-phase entry | O75794 | 2 | 0.25 |
| Protein ERGIC-53 | Forms a specific cargo receptor for the ER-to-Golgi transport of proteins | P49257 | 3 | 0.24 |
| Ribonuclease/angiogenin inhibitor | Regulates RNA turnover | P13489 | 2 | 0.23 |
| Phosphorylcholine transferase A | Regulates phosphatidylcholine synthesis | P49585 | 4 | 0.23 |
| Angiomodulin | Binds IGF-I/-II; stimulates prostacyclin | Q16270 | 9 | 0.21 |
| Collaborator of ARF | Regulates p53-p21-HDM2 pathway | Q9NXV6 | 2 | 0.13 |
| GDP-L-fucose synthetase | Catalyzes 2-step epimerase and reductase reaction of GDP-D-mannose | Q13630 | 2 | 0.06 |
| Probable tubulin polyglutamylase TTLL1 | Modifies α- and β-tubulin, forms glutamate side chains | O95922 | 2 | 0.06 |
| 60S ribosomal protein L22-like 1 | Unknown | Q6P5R6 | 2 | 0.06 |
| **Coiled-coil domain-containing 44** | Translational activator of cytochrome C oxidase 1 | Q9BSH4 | 2 | 0.03 |

**Table S1.**
